# Shear Capacity of C-Shaped and L-Shaped Angle Shear Connectors

**DOI:** 10.1371/journal.pone.0156989

**Published:** 2016-08-01

**Authors:** Farzad Tahmasbi, Shervin Maleki, Mahdi Shariati, N. H. Ramli Sulong, M. M. Tahir

**Affiliations:** 1 Department of Civil Engineering, Sharif University of Technology, Tehran, Iran; 2 Department of Civil Engineering, University of Malaya, 50603, Kuala Lumpur, Malaysia; 3 UTM Construction Research Centre, Faculty of Civil Engineering, Institute for Smart Infrastructure and Innovative Construction, UTM, 81310, Johor Bahru, Johor, Malaysia; Northwestern Polytechnical University, CHINA

## Abstract

This paper investigates the behaviour of C-shaped and L-shaped angle shear connectors embedded in solid concrete slabs. An effective finite element model is proposed to simulate the push out tests of these shear connectors that encompass nonlinear material behaviour, large displacement and damage plasticity. The finite element models are validated against test results. Parametric studies using this nonlinear model are performed to investigate the variations in concrete strength and connector dimensions. The finite element analyses also confirm the test results that increasing the length of shear connector increases their shear strength proportionately. It is observed that the maximum stress in L-shaped angle connectors takes place in the weld attachment to the beam, whereas in the C-shaped angle connectors, it is in the attached leg. The location of maximum concrete compressive damage is rendered in each case. Finally, a new equation for prediction of the shear capacity of C-shaped angle connectors is proposed.

## 1. Introduction

Composite beams are recognized for their high strength and stiffness and reliable structural behaviour. The strength and ductility of shear connectors play a vital role in the design of composite beams. The successful design of shear connectors relies heavily on the existing experimental investigations on the load-slip behaviour of the connector. Many forms of shear connectors are being used in composite beams, however, economical and structural aspects motivates new innovations like C-shaped and L-shaped angle shear connectors. Present knowledge of the load–displacement behaviour and the shear capacity of shear connectors are mainly limited to the data obtained from the experimental push-out or beam tests [1–3]. Experimental tests are expensive and time-consuming option for such investigations and in some cases can even be impractical.

According to the experimental studies on channels by Maleki et al. [1–3] and angle shear connectors by Shariati et al. [4–9], and the similarity of channels and angles (except for one leg), it is concluded that push-out test is an appropriate method to find the load-displacement behavior of C-shaped and L-shaped angle shear connectors. The current research differs from the previous studies in considering different orientation of angle shear connectors and comparing them in strength.

Numerical methods to predict the nonlinear load-slip relationship and the ultimate shear capacity of the shear connectors in composite beams are definitely a valuable option. More so, when the numerical methods are substantiated by accurate experimental results.

Finite element (FE) method has become a powerful tool for the numerical analysis of a wide range of engineering problems. An accurate finite element model permits a considerable reduction in the number of experiments needed for the prediction of structural behaviour.

Nevertheless, in a study of any structural system beyond the elastic range, the experimental phase is essential. Taking into account that FE models should be backed by reliable test results, experimental and numerical studies can complement each other in the investigation of a particular structural phenomenon.

There are limited studies available on the finite element modeling of the push-out specimens. The primary studies are concentrated on stud shear connectors conducted by Nakajima et al. [10] and Lam and El-Lobody [11]. A comprehensive finite element study on the behavior of channel shear connectors was conducted by Maleki and Bagheri [2] and Maleki and Mahoutian [3]. In another finite element analysis of push-out test, Khalilian and Maleki [10] suggested a new equation for prediction of shear strength of C-shaped angle connectors in composite beam. There are some other finite element studies that focused on other types of shear connectors and the composite beams and help in better understanding of the way of finite element analysis in this area [11–14].

The aim of this paper is to develop a finite element model for the angle connectors that can match the results of the experiments with good accuracy. The push-out test arrangement is modelled in FE environment and all linear and nonlinear properties of components are taken into consideration to establish the ultimate strength and load–displacement behaviour of the connector under monotonic loading. The results of the present FE model are compared with push-out tests. Parametric studies using this model are carried out to investigate the effects of variations in concrete strength and connector dimensions. Finally, a new equation for the shear capacity of L-shaped angle connectors is proposed.

## 2. Experimental Program

**Fig 1:**
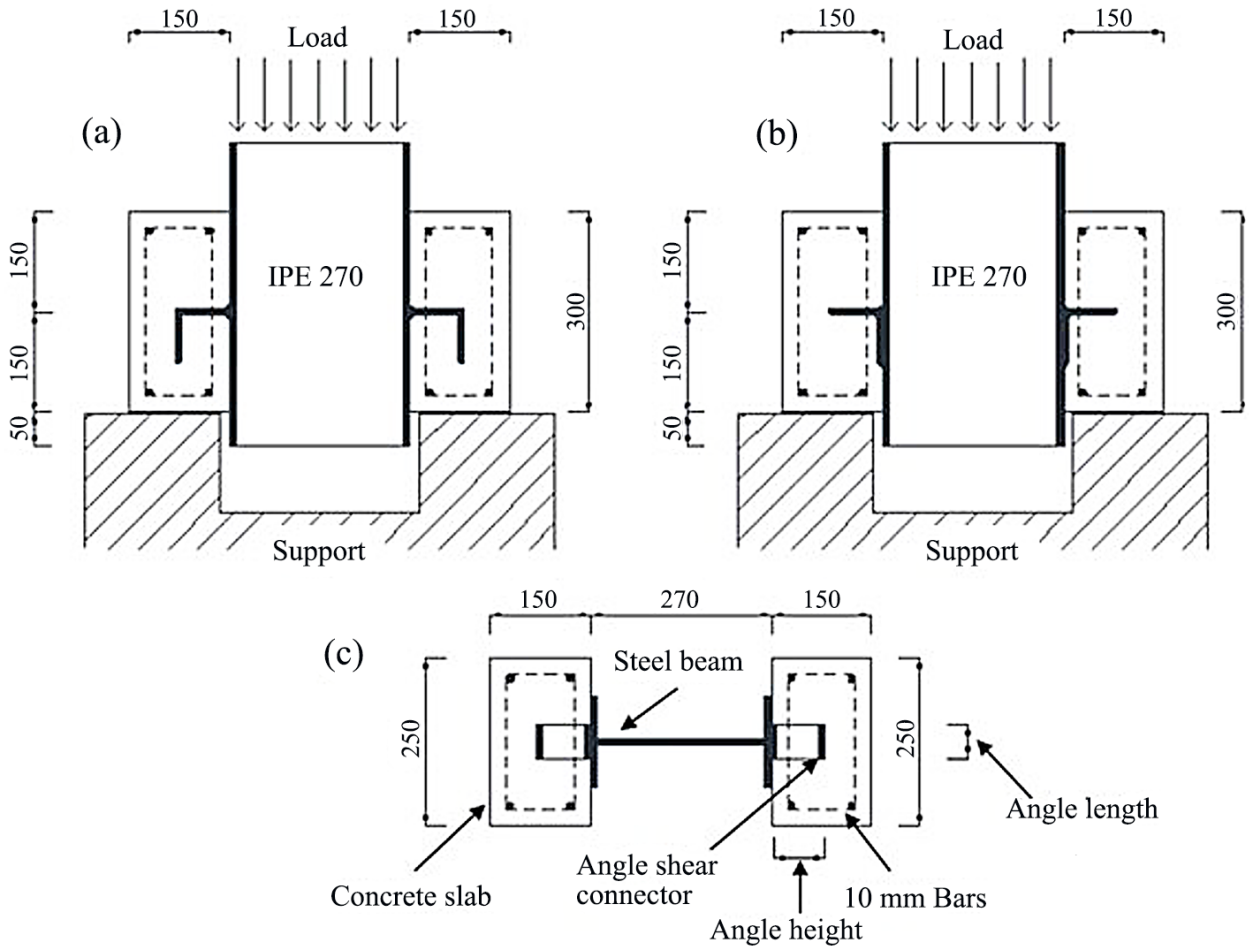
Specimens’ details, (a) Side view of C-shaped angle connectors (MV specimens), (b) Side view of L-shaped angle connectors (MH specimens), (c) Top view.

Eight composite slabs with angle shear connectors are fabricated and tested by the push-out method. The specimens consist of two concrete blocks with embedded tie reinforcement, a steel rolled I-section and two angle shear connectors connecting to the flanges of I-section. The steel I-section is a European IPE270. All structural steels are ST37 grade with nominal minimum yield strength of 240 MPa. The angle shear connectors are welded to the steel beam flange in C-shaped and L-shaped configuration as shown in Fig 1.

Specimens’ designation consists of two letters and a number. The first letter M, stands for one directional monotonic loading. The second letter is V or H corresponding to the C or L-shaped configurations, respectively. These letters are followed by the angle size in mm.

In each configuration, four specimens were tested consisting of different angle sizes and lengths as indicated in Table 1.

**Table 1:** Properties of specimens.

Specimen	Angle height (mm)	Angle length (mm)	Angle thickness (mm)	Concrete strength (MPa)
MV60	60	50	6	29.45
MV80	80	50	8	22.43
MV100	100	50	10	25.42
MV*80	80	100	8	31.07
MH60	60	50	6	29.25
MH80	80	50	8	29.5
MH100	100	50	10	24.97
MH*80	80	100	8	27.92

The equal leg angles used are of sizes commonly incorporated in composite beams. Specimens were chosen so as to study the parameters that influence the shear strength of angle shear connectors. Parameters include height, length, thickness and angle position. Fig 1 shows that the thickness of the concrete slab in all specimens was 150 mm. The width and height of the slabs were 250 and 300 mm, respectively. The diameter of steel longitudinal and transverse reinforcements embedded in the slabs was 10 mm. The angle shear connectors were welded to the flange of steel beam with 7 mm fillet welds.

The materials used in concrete blocks were Portland cement, coarse aggregate, river sand and water. The weight ratios of cement, water, sand and gravel used were 1, 0.42, 2.75 and 1.75, respectively.

Load was applied on each specimen with a universal testing machine of 1000 kN capacity using displacement control with a rate of 0.1mm/s for all specimens. Monotonic loading was continuously applied until the specimen clearly begun to fracture and fail. The load-displacement results of each specimen were automatically plotted by the hardware attached to the universal testing machine. The displacement measured is the relative displacement between the top of the steel beam and the bottom of concrete block in each time step.

All specimens failed in concrete crushing/splitting mode except for MV60 and MH60 which had connector failure.

## 3. Analytical Study

The finite element program ABAQUS [15] is used to simulate the push-out tests. This software is able to consider nonlinear material behavior in steel and concrete, nonlinear geometry as large displacement and tensile and compressive damage in concrete. The static implicit analysis method is employed with stepwise displacement loading.

### 3.1. Finite element model and mesh

**Fig 2:**
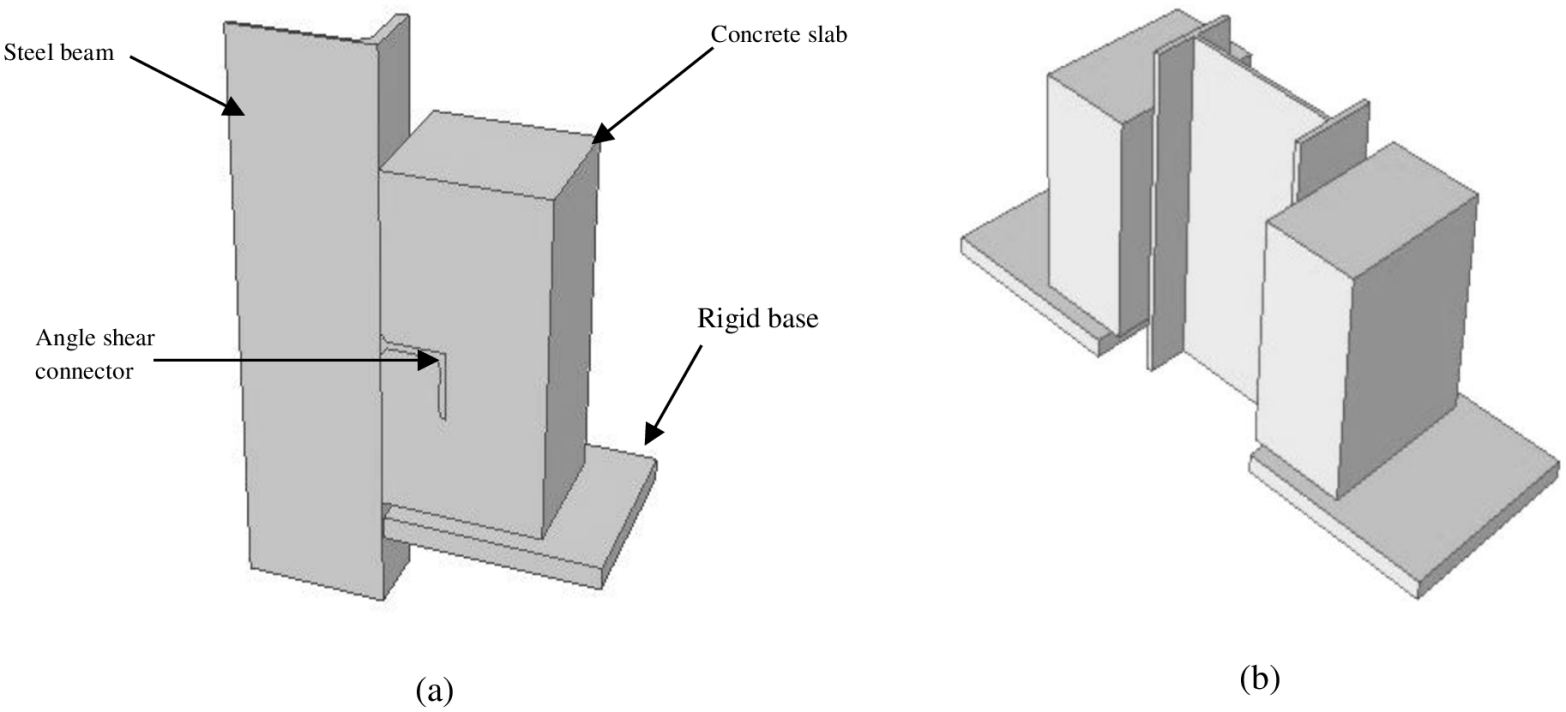
Model of push-out test specimen (a) A quarter of push-out test specimen & (b) Full view of push-out test specimen.

To achieve accurate results from the finite element program, it is crucial to model all the details of the push-out specimen. The FE model has five parts: concrete slab, steel beam, shear connectors, rebar and rigid base. Since specimen is symmetric, only a quarter of specimen is simulated (Fig 2a). The actual full view of the push-out test specimen is shown in Fig 2b. In modelling of shear connectors the attachment weld has been created in the same part.

Nguyen et al. [16] investigated the use of a cohesive layer between the steel beam and concrete. They concluded that the influence of the layer is limited to initial stiffness and does not affect the ultimate strength and displacement. Hence, to reduce the analysis time, the cohesive layer is not considered here.

**Fig 3:**
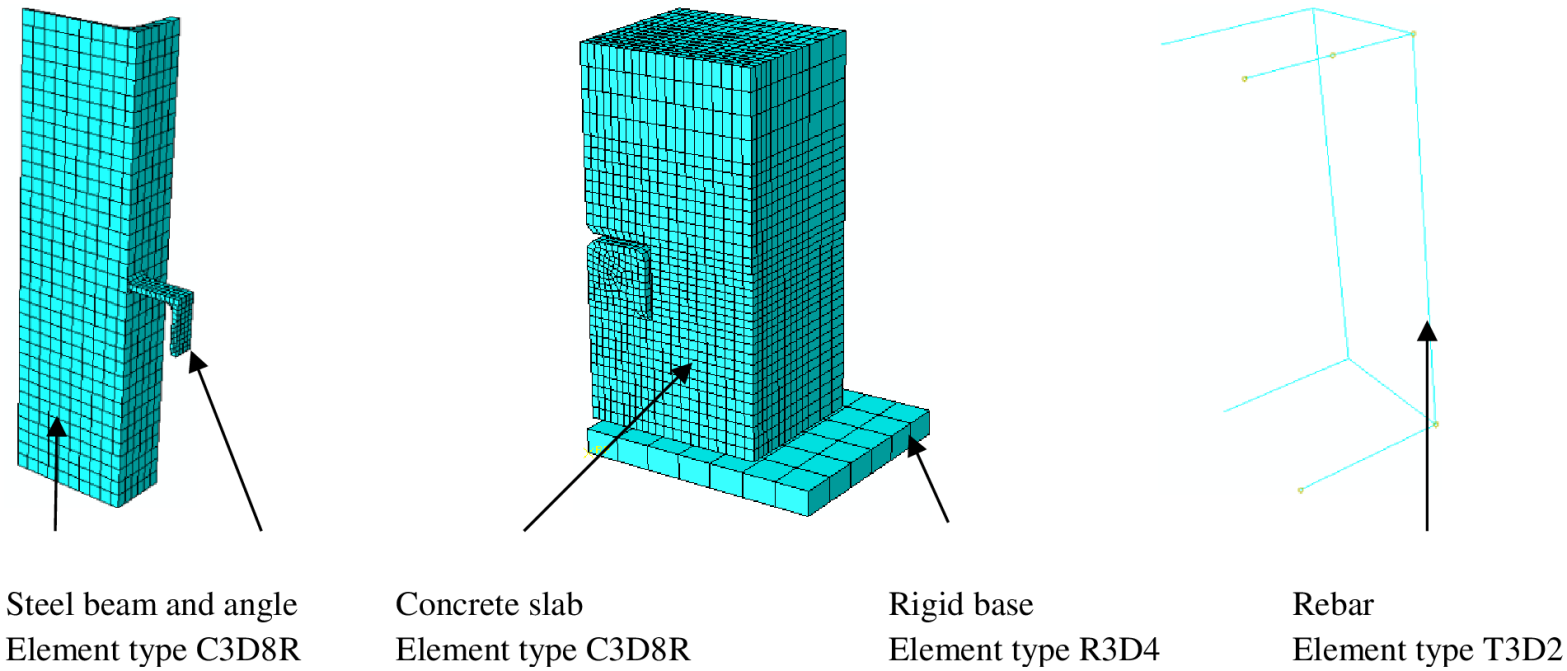
Finite element type and mesh.

The concrete block, steel beam and shear connectors are meshed with solid element C3D8R. This element type is an 8-node brick element with reduced stiffness. Each node has three translational degrees of freedom (DOF). This element can be employed for nonlinear analysis containing contact, large deformation, plasticity and damage. To decrease the analysis time, a coarse mesh is utilized as an overall size and a fine mesh is employed at the critical zones such as around the interface between concrete and shear connector. To mesh all parts of specimen in an orderly way, the specimen was partitioned. The rigid base is modelled using the rigid element R3D4 and for the reinforcement in the concrete block the truss element T3D2 is used. The finite element type and mesh of the specimen is shown in Fig 3.

### 3.2. Contact interaction and constraint conditions

In reality, compressive forces act in physical contact surfaces between the shear connector and surrounding concrete, otherwise, surfaces in contact separate instantly. There are also frictional forces acting on the surfaces of these materials. Hence, in simulation, the contact behaviour must be used properly to model this interaction. In the FE model, surface to surface contact between the concrete block and shear connector is employed. In other words, all surfaces of shear connector which is in contact with the surrounding concrete have been modelled with contact surfaces. Normal to the surface contact is defined as hard contact between surfaces, not allowing the penetration of surfaces into each other. The penalty contact method of ABAQUS is used for tangential behavior. The coefficient of friction is set as 0.45. The concrete block is assumed to be the master surface. Embedded regions are used for simulation of rebar inside the concrete block.

Note that, in the push-out experiment, the steel beam flange surface contacting the concrete slab is usually greased to reduce friction. Therefore, frictionless contact is used at the concrete to steel beam flange interface. In the normal direction, hard contact is assumed.

To impede slipping between the steel beam and the shear connector, the joints at the contact surfaces of the two components are connected via the tie constraint.

**Fig 4:**
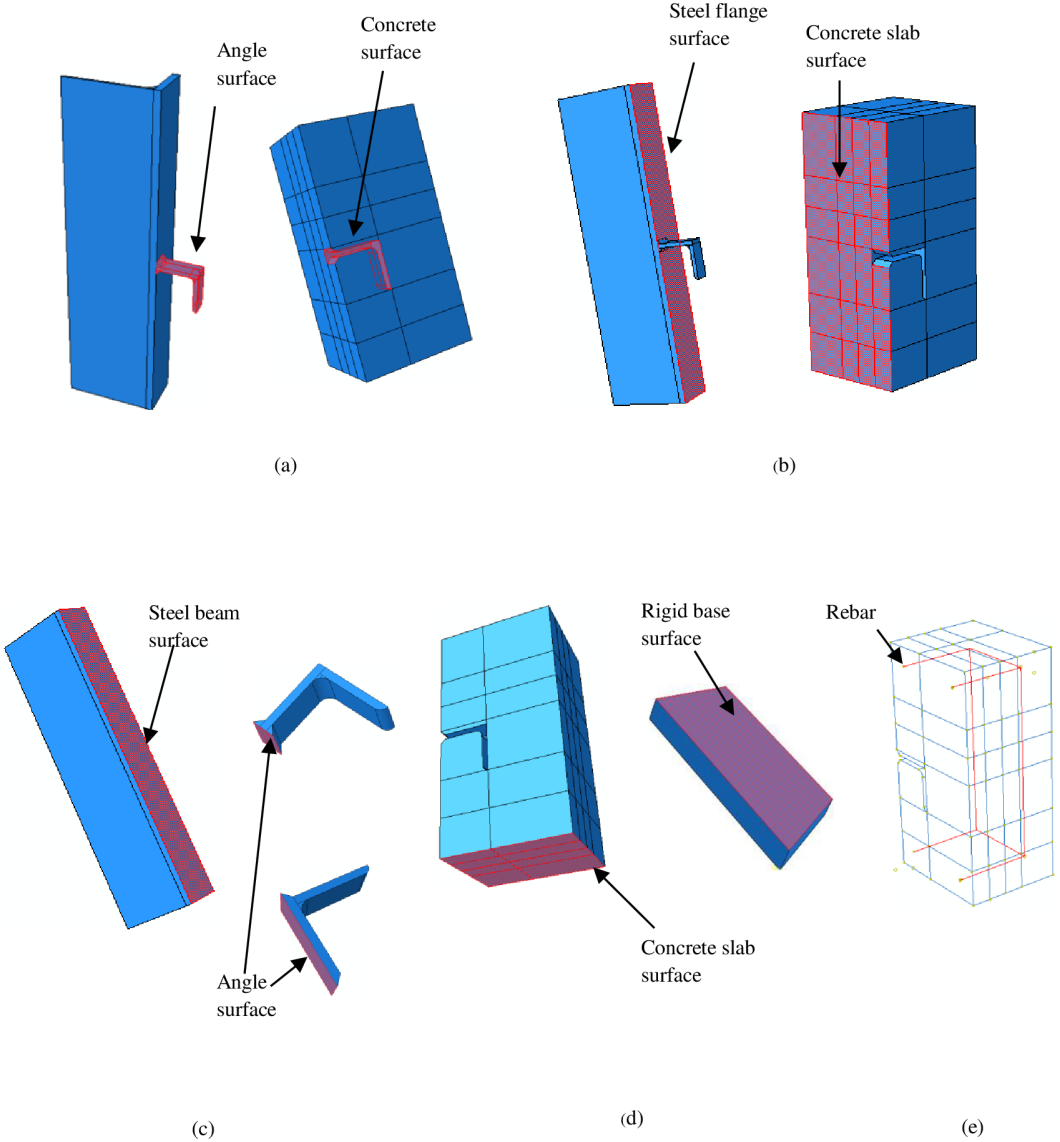
Contact interaction and constraint conditions surfaces. A, Surfaces in contact interaction between angle and concrete. B, Surfaces in contact interaction between steel flange and concrete. C, Surfaces in tie constraint between steel flange and angle. D, Surfaces in contact interaction between rigid base and concrete. E, Rebar embedded in concrete slab.

The contact between concrete slab and rigid base is simulated similar to shear connector and concrete contact, using a tangential friction coefficient of 0.6 and hard contact in the normal direction. The rigid base is considered as the master surface. Contacts interactions and constraint conditions are shown in Fig 4.

### 3.3. Loading and boundary conditions

**Fig 5:**
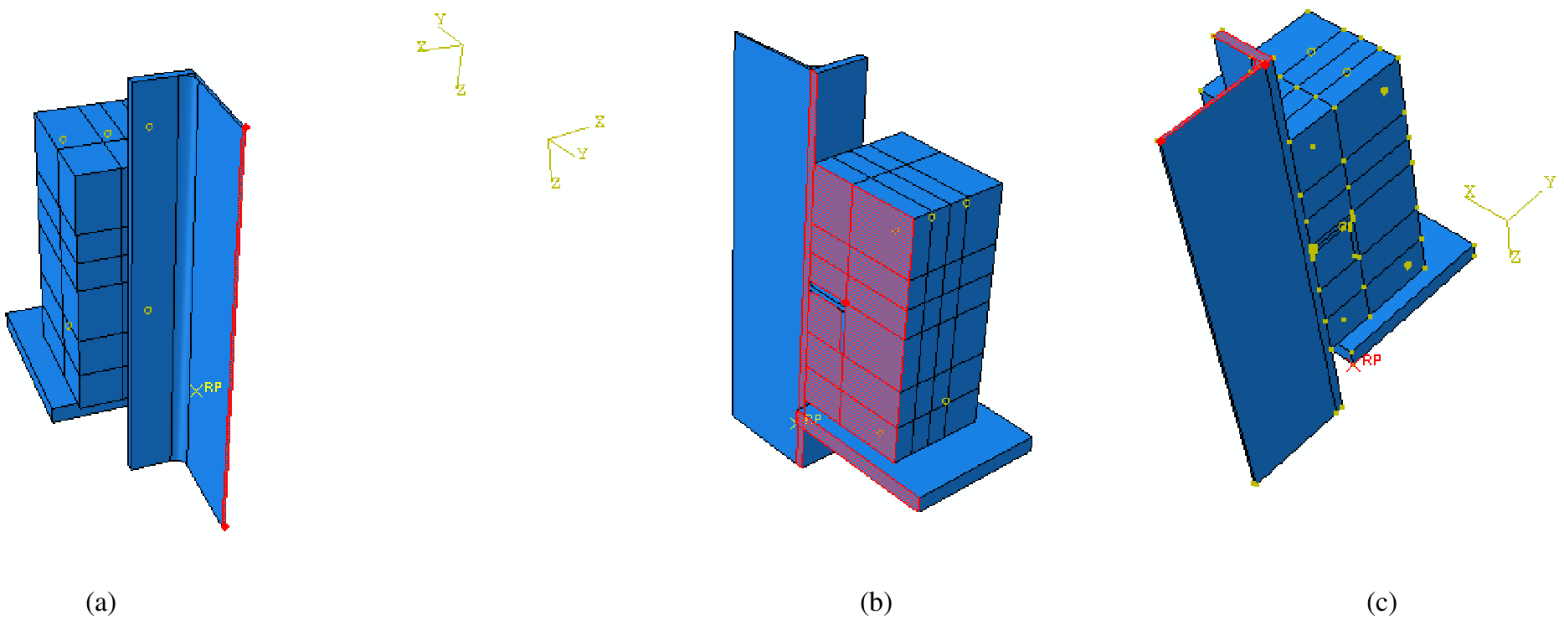
Boundary conditions and loading surface. A, Y-axis symmetric boundary condition. B, X-axis symmetric boundary condition. C, Rigid base boundary condition and loading surface on top.

Since push-out test specimens are symmetric, only a quarter of specimen is modelled for the analytical study. The symmetric boundary conditions are applied to the surfaces at the symmetric planes of the specimen as shown in Fig 5(a) and 5(b). The rigid base supports the assembly without any movement. Hence, all DOF of the rigid base are fixed as shown in Fig 5(c). In this study, displacement control method is used for loading. The load is applied to the top of steel beam (Fig 5(c)). At the beginning of the analysis, the applied displacement is set to zero and then the displacement is increased linearly according to amplitude function.

### 3.4. Material properties

#### 3.4.1. Mechanical properties of concrete

The concrete damaged plasticity model (CDP) available in ABAQUS is employed to model the concrete damage. This material model is based upon two main failure mechanisms of concrete which are tensile cracking and compressive crushing. This is adequate for simulating materials having different behaviours in tension and compression. The initial Young’s modulus of elasticity of concrete is estimated using EC2 [17] provisions as given in Eq 1 below.

$$E_{cm}=22.{\left(\frac{f_{cm}}{10}\right)}^{0.3}$$ (1)

Where:

*E*_*cm*_ = concrete modulus of elasticity (GPa)

*f*_*cm*_ = mean value of concrete compressive cylinder strength at 28 days (MPa)

Poisson’s ratio of concrete is assumed to be 0.2. A normal weight concrete of density 2400 kg/m^3^ is presumed for all concrete grades.

**Fig 6:**
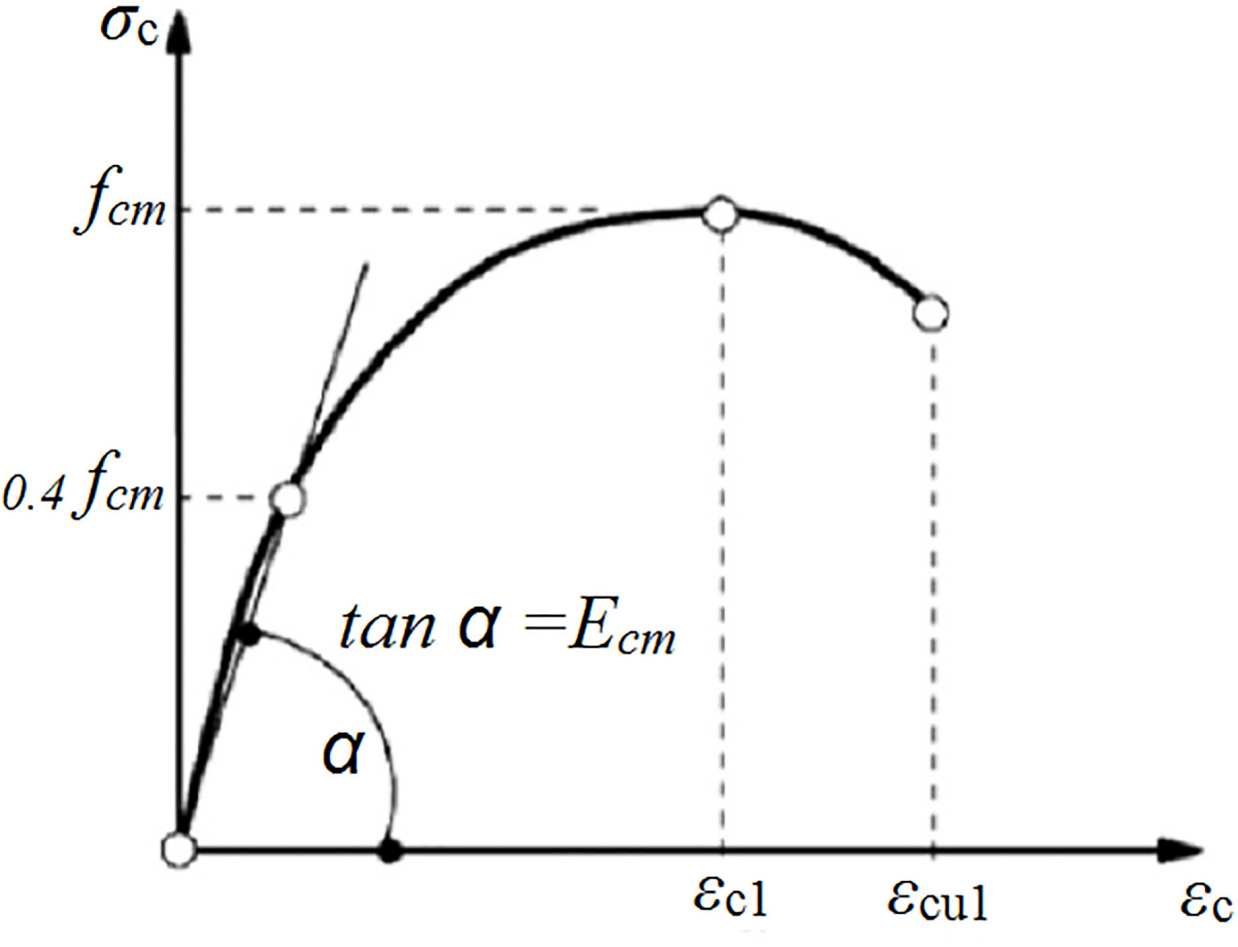
Uniaxial stress-strain curve of concrete EC2 [17].

In this investigation, the dilation angle for concrete plasticity is assumed to be 40 degrees, similar to Qureshi et al. [18] work. Other plasticity parameters such as, K, eccentricity, the ratio of biaxial compressive strength to uniaxial compressive strength *f*_bo_/*f*_co_ are assumed as 0.666, 0.1 and 1.16, respectively. Uniaxial stress-strain curve of concrete in compression is shown in Fig 6.

The concrete acts linearly up to a stress of 0.4f_cm_.The strain *ε*_c1_ associates with f_cm_ is 0.0022, as recommended by EC2 [17]. The nonlinear compressive stress-strain relationship of concrete beyond the 0.4f_cm_ stress is calculated from Eq 2 per EC2 [17].

$$\sigma_{c}=\left(\frac{k\eta -\eta^{2}}{1+(k-2)\eta }\right)\;f_{cm}$$ (2)

Where:

$$k=1.05E_{cm}\times \frac{\varepsilon_{c1}}{f_{cm}}\;\&\;\eta =\frac{\varepsilon_{c}}{\varepsilon_{c1}}$$

**Fig 7:**
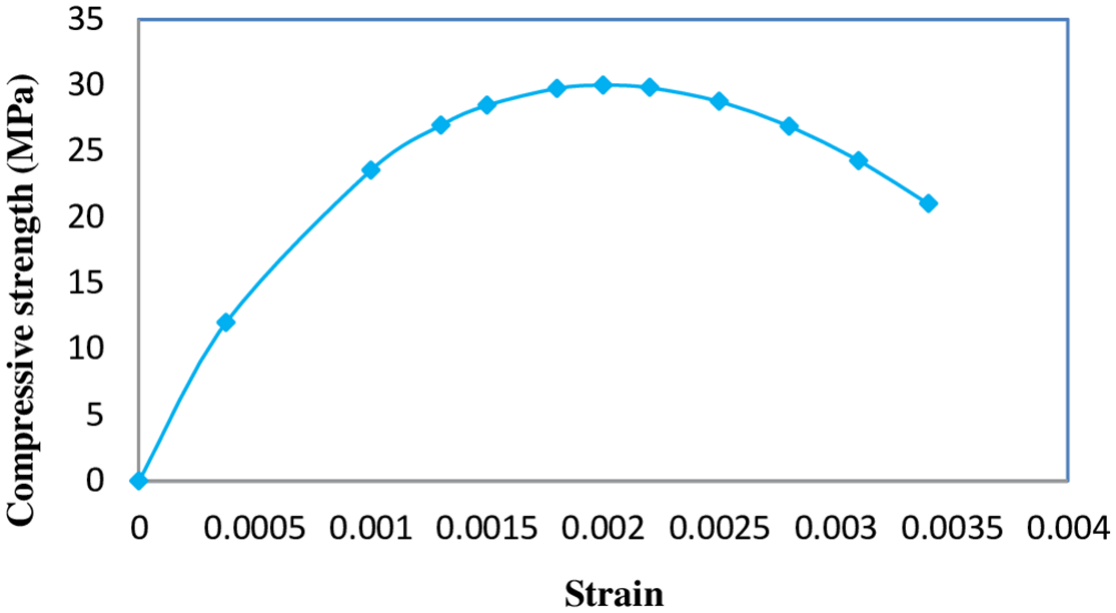
Un-axial stress-strain curve for 30 MPa concrete.

The ultimate strain *ε*_cu_of concrete at failure according to EC2 [17] is equal to 0.0035. The uniaxial stress-strain curve of concrete in compression having a mean compressive cylinder strength of 30MPa is shown in Fig 7.

It is worth noting that in ABAQUS, for simulating the concrete damage plasticity model (CDP), an inelastic strain (*ε*_in_) is utilized as follows,

$$\varepsilon_{el}=\frac{0.4f_{cm}}{E_{cm}}$$ (3)

$$\varepsilon_{in}=\varepsilon_{c}-\varepsilon_{el}^{}$$ (4)

Where εelis the elastic strain and εc is the strain in concrete in the plastic range. The CDP model assumes that the concrete behaviour beyond the peak stress (f_cm_) is accompanied by damage. This includes a reduction in stiffness from the initial elastic modulus E_cm_ (Fig 6). This reduction is assumed to be linear with respect to stress as indicated by the following Eq 20:

$$d_{c}=1-\frac{\sigma_{c}}{f_{cm}}$$ (5)

Where dc is the concrete damage parameter ranging from zero to one, the former indicating compression failure.

**Fig 8:**
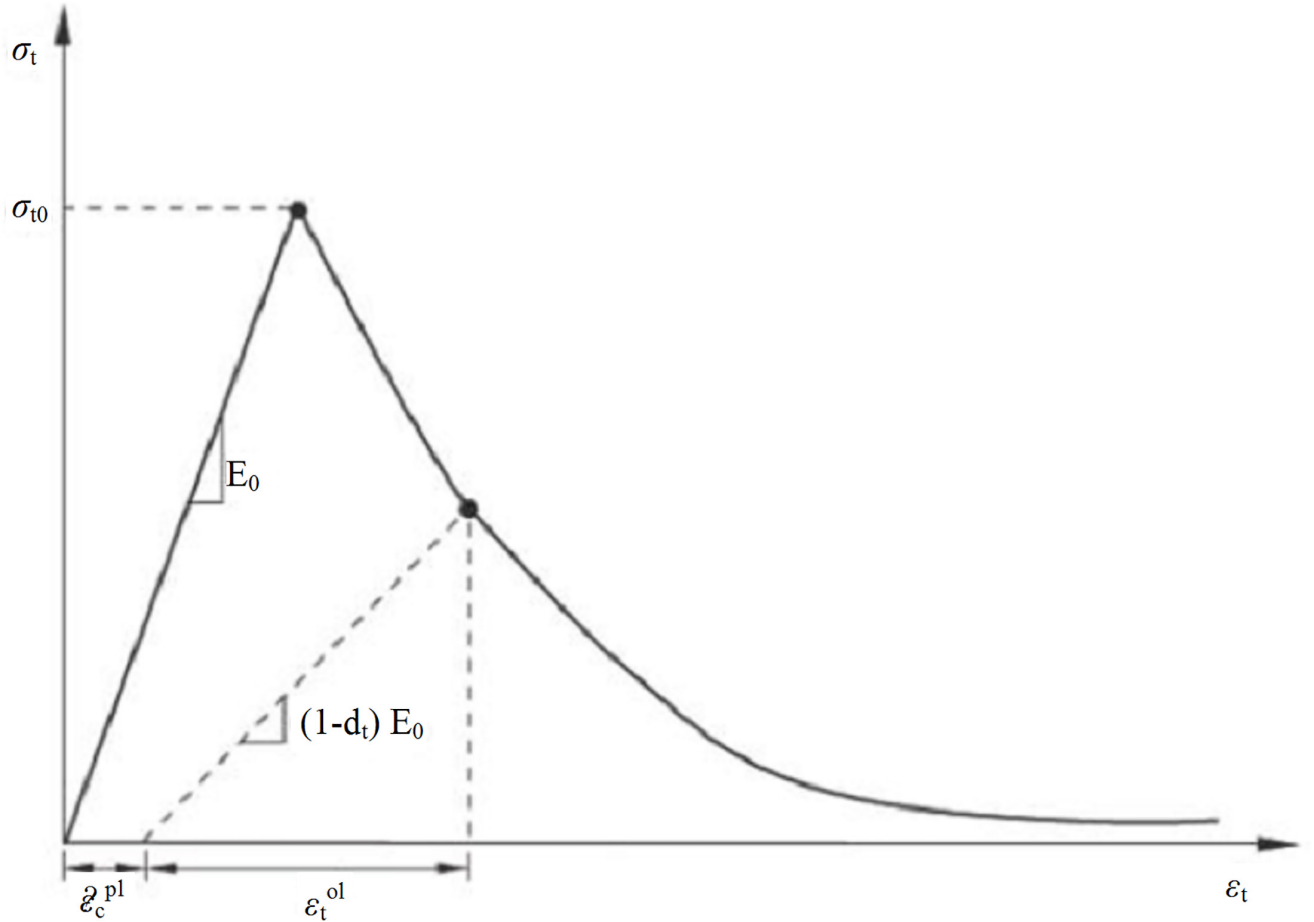
Stress-strain curve of tensile behaviour of concrete [15].

In tension, it is assumed that concrete tensile stresses increase linearly with the rising of strain. This is accompanied by the development of small cracks in concrete. After the concrete cracks, the behaviour is defined by softening of stress–strain response as cracks develop wider. It is assumed that the stress-strain curve exponentially decreases to zero stress. In Fig 8 the tensile stress-strain behaviour of concrete is shown.

**Fig 9:**
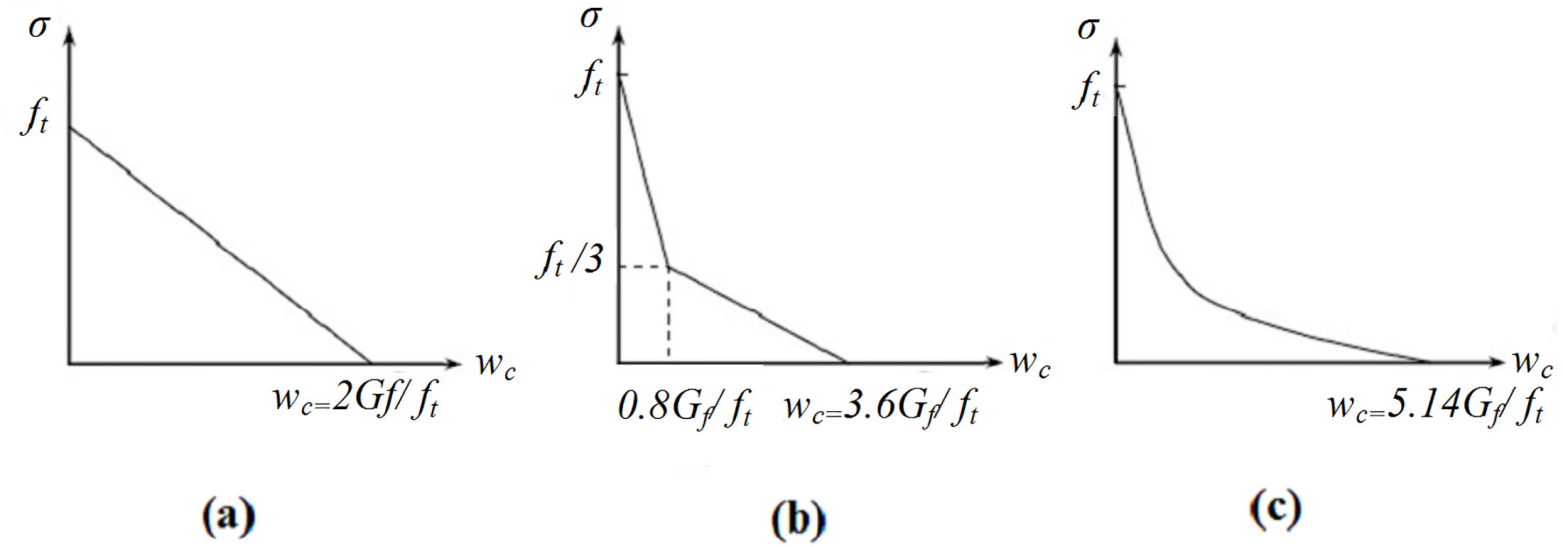
Linear (ABAQUS manual)(a), Bilinear [19] (b) and exponential [20] tension softening model [15] (c).

To define the softening behaviour of concrete when none or small amount of reinforcement is present, the utilization of tension stiffening approach is recommended. The use of stress-strain curve has shown mesh sensitivity. For solving this problem, the fracture energy approach of Hillerborg et al. [19] is utilized. The simplest method is to define tension softening model via linear approximation, in which the linear loss of strength happens after cracking, as presented in Fig 9(a). Further improvement can be achieved by using a bilinear function developed by Hillerborg [19], as demonstrated in Fig 9(b). A more effective technique of defining tension softening is to apply an exponential expression, as experimentally derived by Cornelissen et al. [20] and is illustrated in Fig 9(c). Therefore, in this research, the latter approach with exponential function is used.

Accordingly, the tension stress with respect to the cracking displacement can be characterized by Eq 6 [20].

$$\sigma_{\text{t}/}{\text{f}}_{\text{t}\;=\;}\text{f}\left(\text{w}\right)-\left(\text{w}/{\text{w}}_{\text{c}}\right)\text{f}\left({\text{w}}_{\text{c}}\right)$$ (6-a)

$$\text{f}\left(\text{w}\right)=\left[1+{\left({\text{c}}_{1}\text{w}/{\text{w}}_{\text{c}}\right)}^{3}\right]\text{exp}\left(-{\text{c}}_{2}\text{w}/{\text{w}}_{\text{c}}\right)$$ (6-b)

Where:

*f*_t_ = tensile strength of concrete

*σ*_t_ = tensile stress of concrete

w = crack opening, mm

*w*_c_ = crack opening at which stress cannot be transferred (assumed 0.35 mm in this investigation)

c_1_ = material constant and c_1_ = 3.0 for normal density concrete

c_2_ = material constant and c_2_ = 6.93 for normal density concrete

The tensile damage parameter can be characterized by Eq 7.

$${\text{d}}_{\text{t}}=1-\sigma_{\text{t}}/{\text{f}}_{\text{t}}$$ (7)

**Fig 10:**
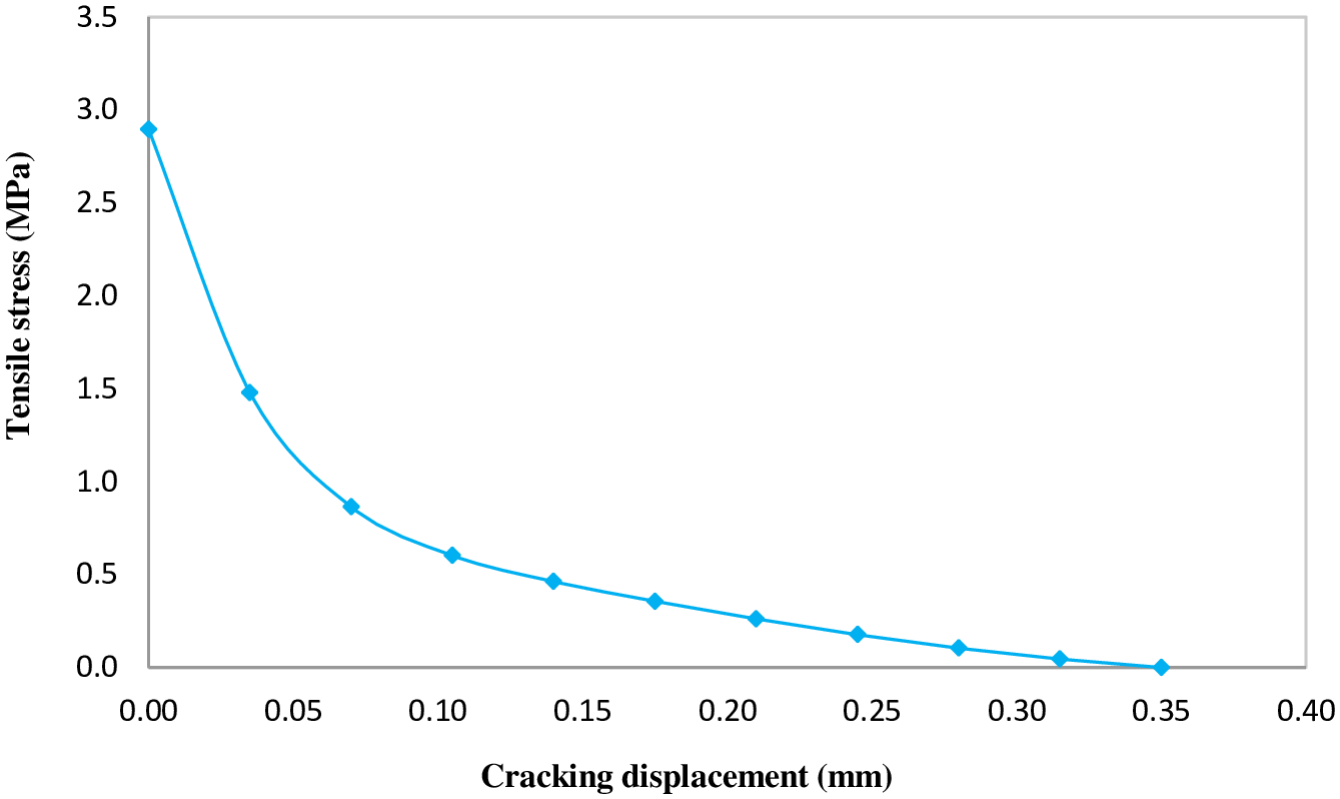
Tensile stress versus cracking displacement curve.

**Fig 11:**
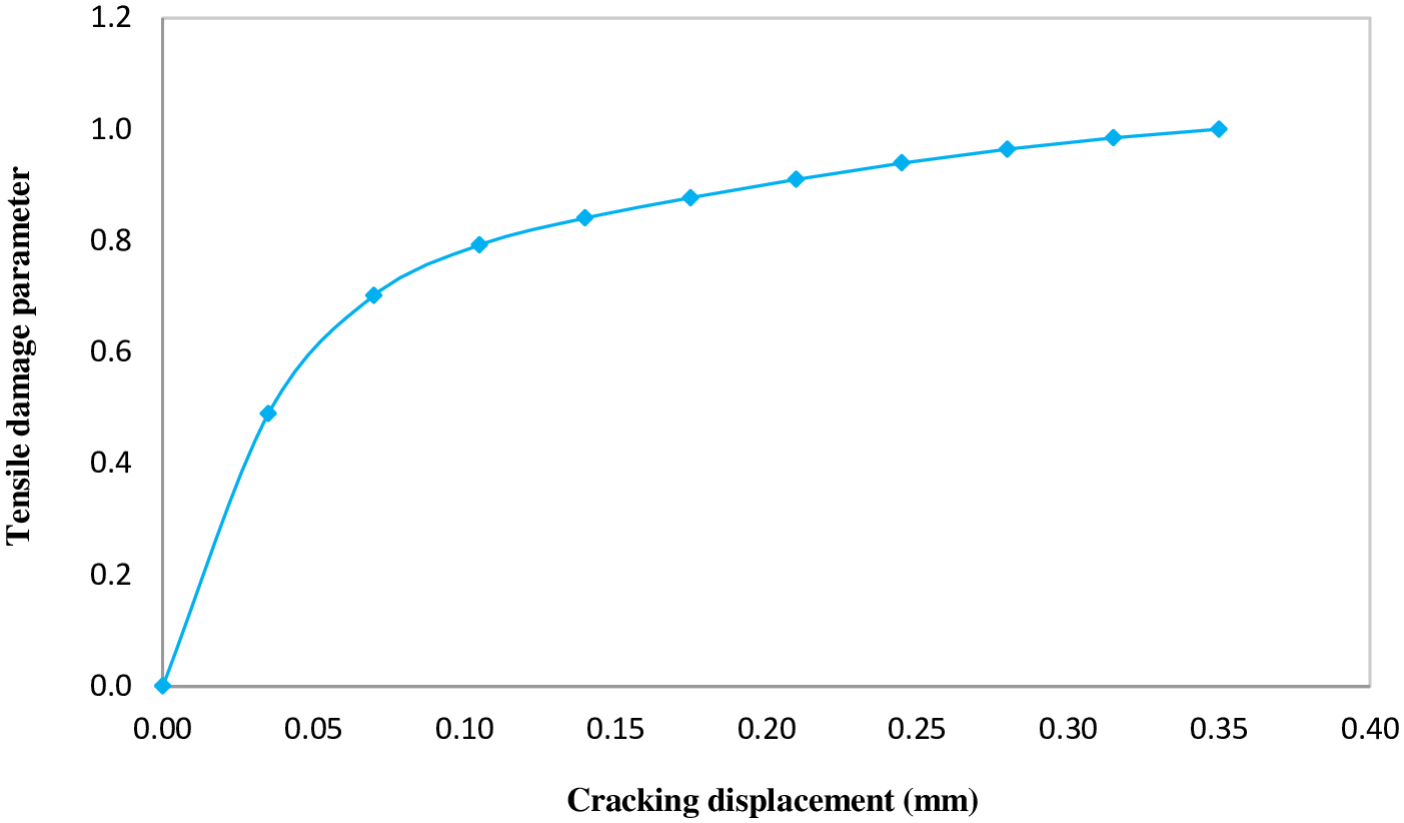
Tensile damage parameter versus cracking displacement.

Tensile damage parameter varies from zero when undamaged, to one in the fully damaged state. Concrete tensile strength is assumed to be one-tenth of the compressive strength. The tensile stress-cracking displacement curve and tensile damage parameter-cracking displacement are presented in Figs 10 and 11, respectively.

#### 3.4.2. Mechanical properties of steel

**Fig 12:**
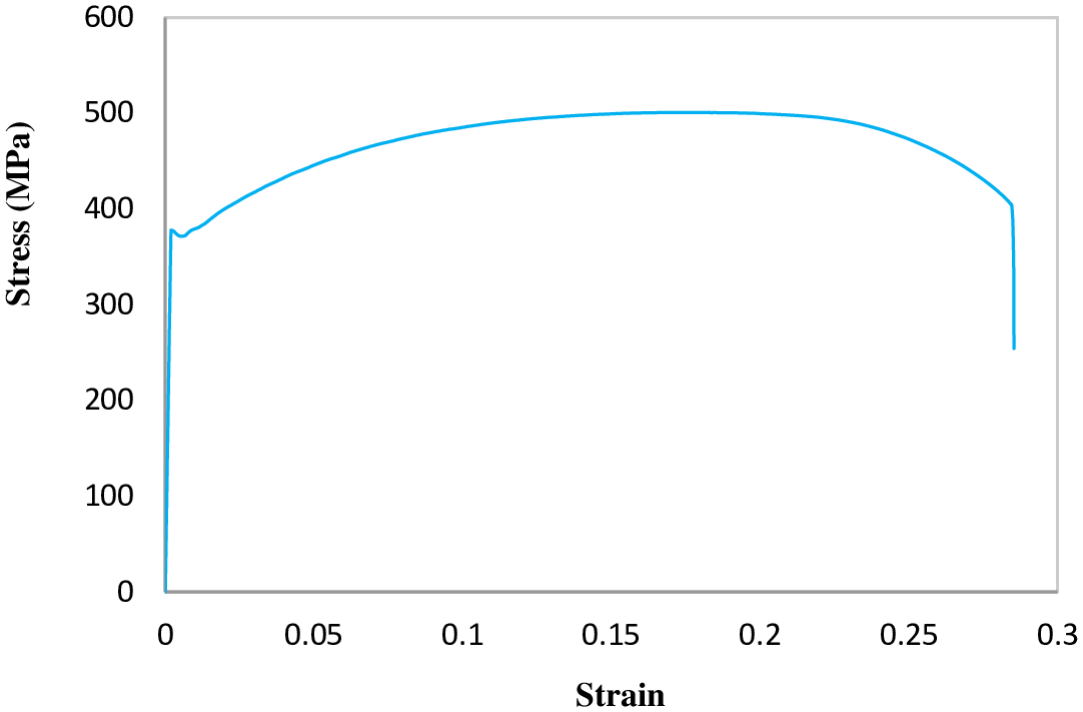
Uniaxial stress-strain curve for steel.

The engineering stress-strain relationship of steel angle is obtained by tensile test as shown in Fig 12. However, in ABAQUS the true stress-strain relationship must be input. Therefore, Eqs 8 and 9 are used to obtain the true stress-strain values. Moreover, the steel angle material is modelled as elasto-perfectly plastic material for simplicity. This is justified by knowing that most damage occurred in the concrete during the tests. Table 2 shows the true stress-strain data for the angle shear connectors as input in ABAQUS.

**Table 2:** Steel properties.

Nominal strain	Nominal stress (MPa)	True stress (MPa)	True strain	ABAQUS input	
				Stress (MPa)	Plastic strain
0.0000	0.00	0.00	0.0000	0.00	0.0000
0.0018	377.68	378.36	0.0018	378.36	0.0000
0.2833	500.73	642.59	0.2494	642.59	0.2464

$$\sigma_{\text{true}}\;=\;\sigma_{\text{nom}}\left(1+\varepsilon_{\text{nom}}\right)$$ (8)

$$\varepsilon_{\text{ln}}{}^{\text{pl}}=\text{ln}\left(1+\varepsilon_{\text{nom}}\right)-\sigma_{\text{true}/}{}_{\text{E}}$$ (9)

The modulus of elasticity of steel used in the models is 208 GPa and the Poisson’s ratio and density are assumed to be 0.3 and 7850 *kg/m*^*3*^ respectively.

## 4. Results and Discussions

### 4.1. Comparison of experimental results and FE simulation for C-shaped angle connectors

**Fig 13:**
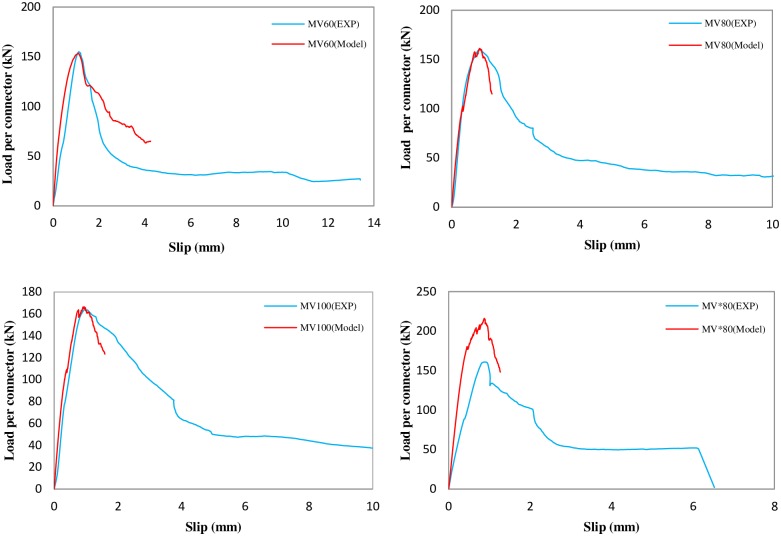
Comparison of experimental results and finite element simulation for C-shaped angle connectors. Concrete f’_c_ = 29.45,22.43,31.07,25.42MPa (from top left clockwise).

**Fig 14:**
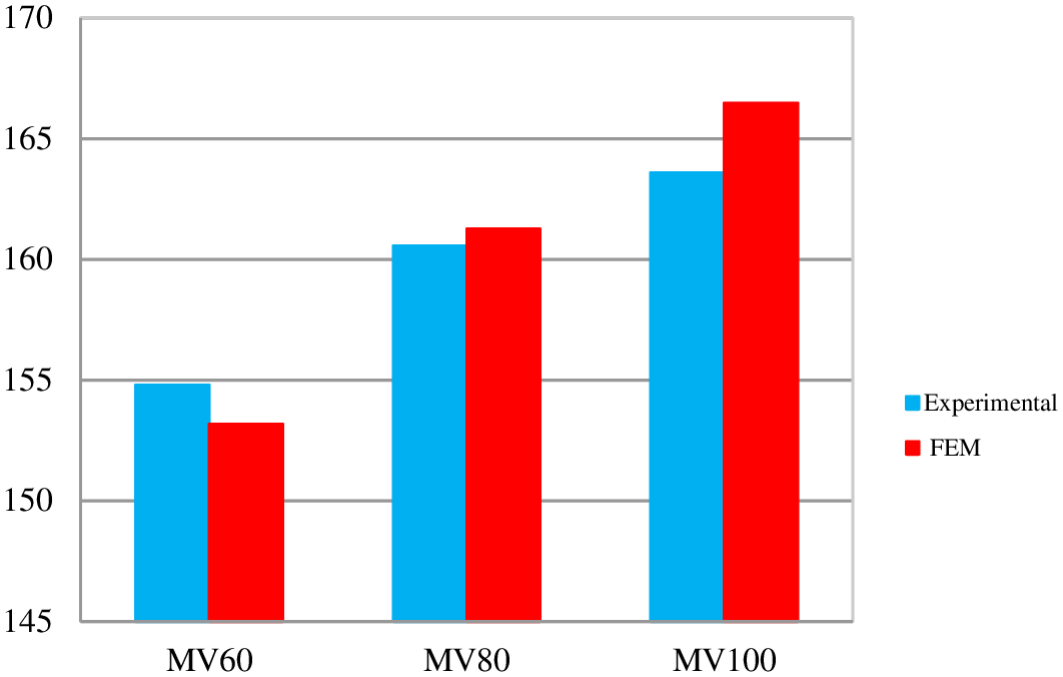
Comparison of the shear strength capacities of C-shaped angle connectors obtained from FE analyses and experiments.

In this section, the load–slip curves for specimens from laboratory tests are compared against the finite element simulations for the C-shaped angle connectors. Fig 13 shows this comparison. Note that, the concrete damage plasticity model terminates the FE analysis earlier than the test. It is seen that FE simulation predicts the peak strength of the connector with good accuracy, except for the MV*80 specimen. The test results for MV*80 was erroneous due to asymmetric failure of one side of the push-out specimen. Therefore, the FE result for this case is more reliable. A more precise comparison of the shear strengths is given in Fig 14. This figure is shown as Figure A in S1 File. The maximum discrepancy between the test results and FE simulations is about 2%.

The FE results also confirm that increasing the length of the connector from 5 cm (in MV80) to 10 cm (in MV*80) increases the strength by about 40%.

### 4.2. Comparison of experimental results and FE simulation for L-shaped angle connectors

**Fig 15:**
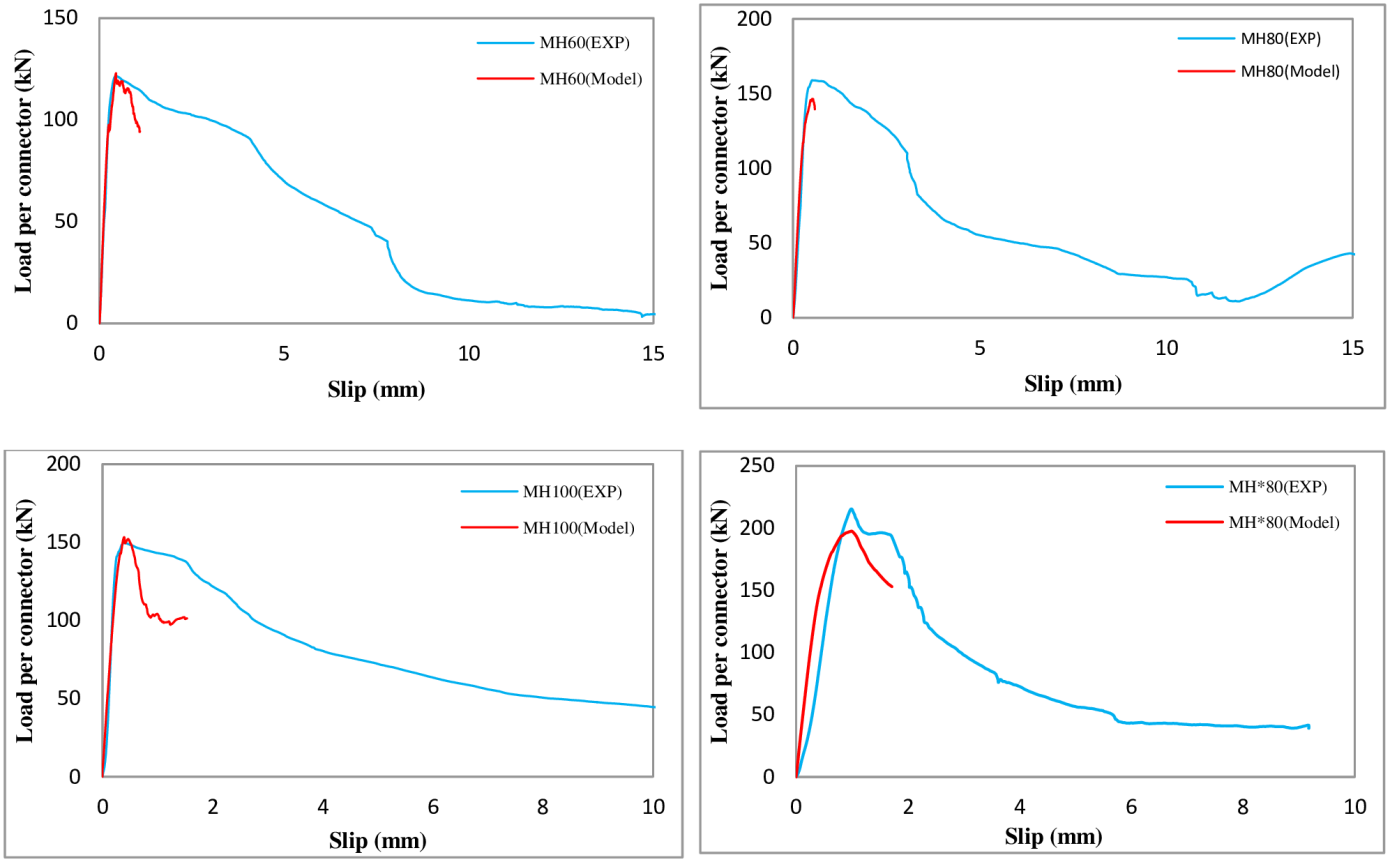
Comparison of experimental results and finite element simulation for L-shaped angle connectors. Concrete f’_c_ = 29.25,29.5,27.92,24.97MPa (from top left clockwise).

**Fig 16:**
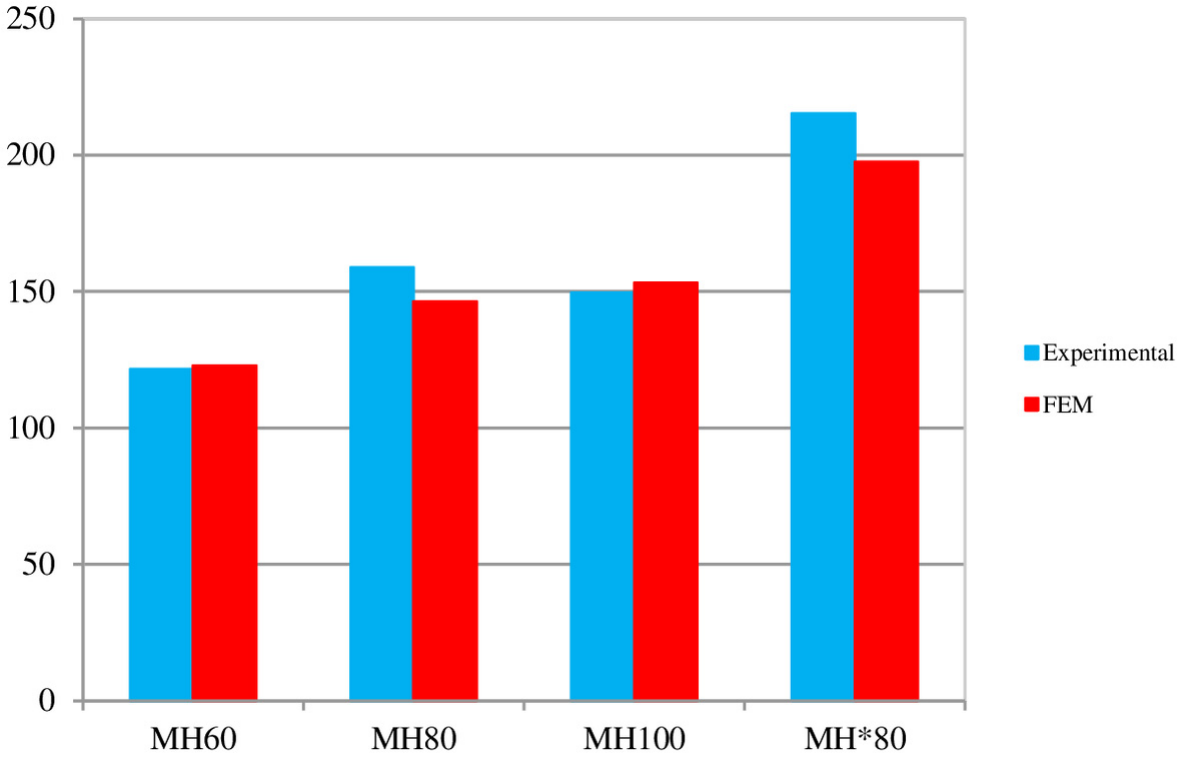
Comparison of the shear strength capacities of L-shaped angle connectors obtained from FE analysis and experimental.

In Fig 15, the load–slip curves for specimens from laboratory tests are compared against the finite element simulations for the L-shaped angle connectors. Fig 16 which is shown as Figure B in S2 File compares the predicted shear capacities in detail and shows a difference of less than 10% in the worst case. The increase in length of the connector from 5 cm to 10 cm, has increased the shear capacity by 35% in MH*80 specimen.

## 5. Parametric Study

### 5.1. General

The results of the previous section indicate that the FE model is well calibrated against the test results. The FE simulation and test results indicate that increasing the angle height decreases the shear capacity of the C-shaped connectors and increases the capacity of L-shaped connectors and for both connectors, increasing the length of shear connectors increases their shear strength.

**Fig 17:**
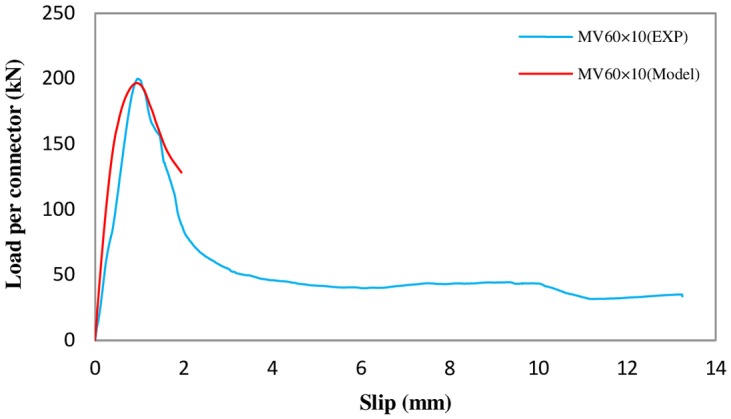
Comparison of parametric analysis of experimental result and finite element simulation for MV60×10.

The other parameter of interest is the effect of angle thickness on the shear strength of connectors. It was suspected from the experimental test results that the shear strength of the C-shaped connector is proportional to the square root of the angle thickness. Therefore, FE model of the MV-60 is reconstructed with 10 mm angle thickness and the results are compared with test results of MV-60 with square root adjustment of √(10/6). The comparison is shown in Fig 17. As it can be seen, an excellent agreement is achieved. This indicates that the FE analysis is in agreement with the assumption of square root of the thickness proportionality.

**Fig 18:**
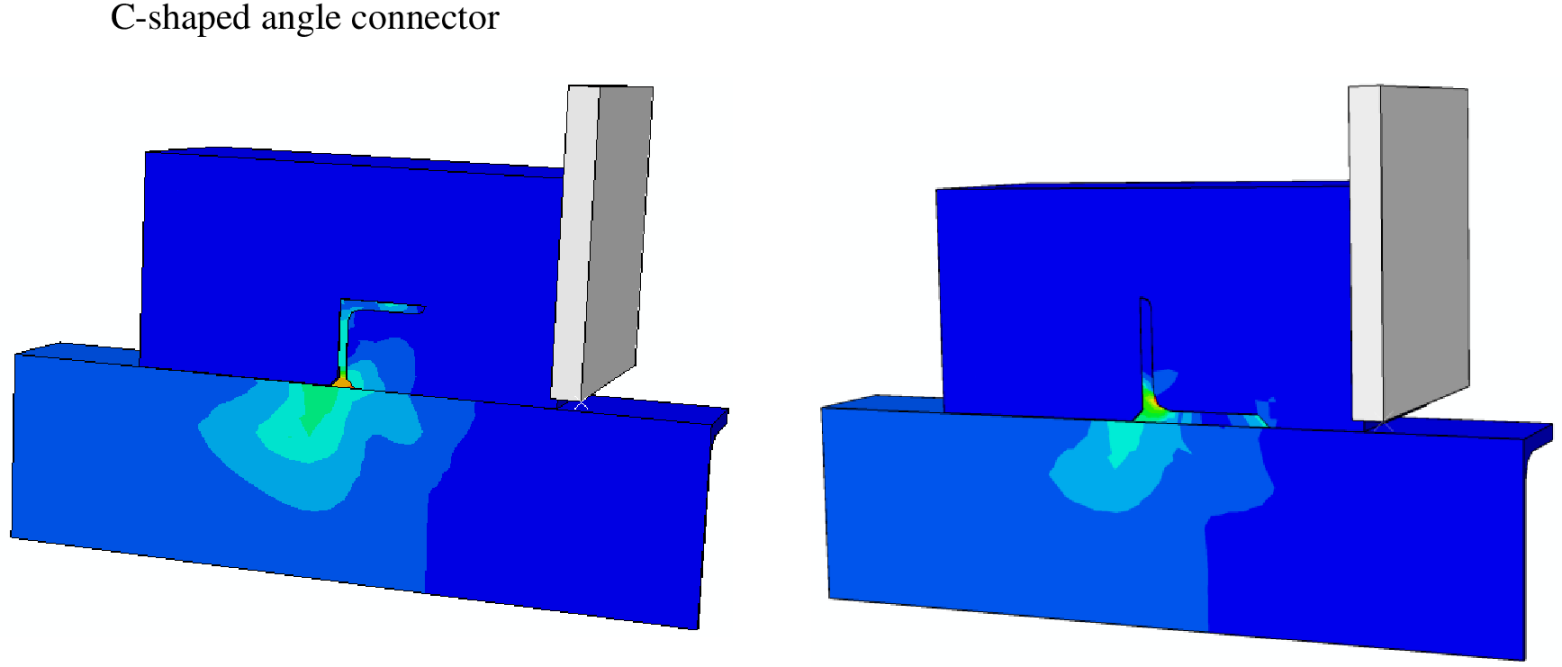
Stress distribution calculating from ABAQUS analysis for one of C-shaped and L-shaped angle connector.

**Fig 19:**
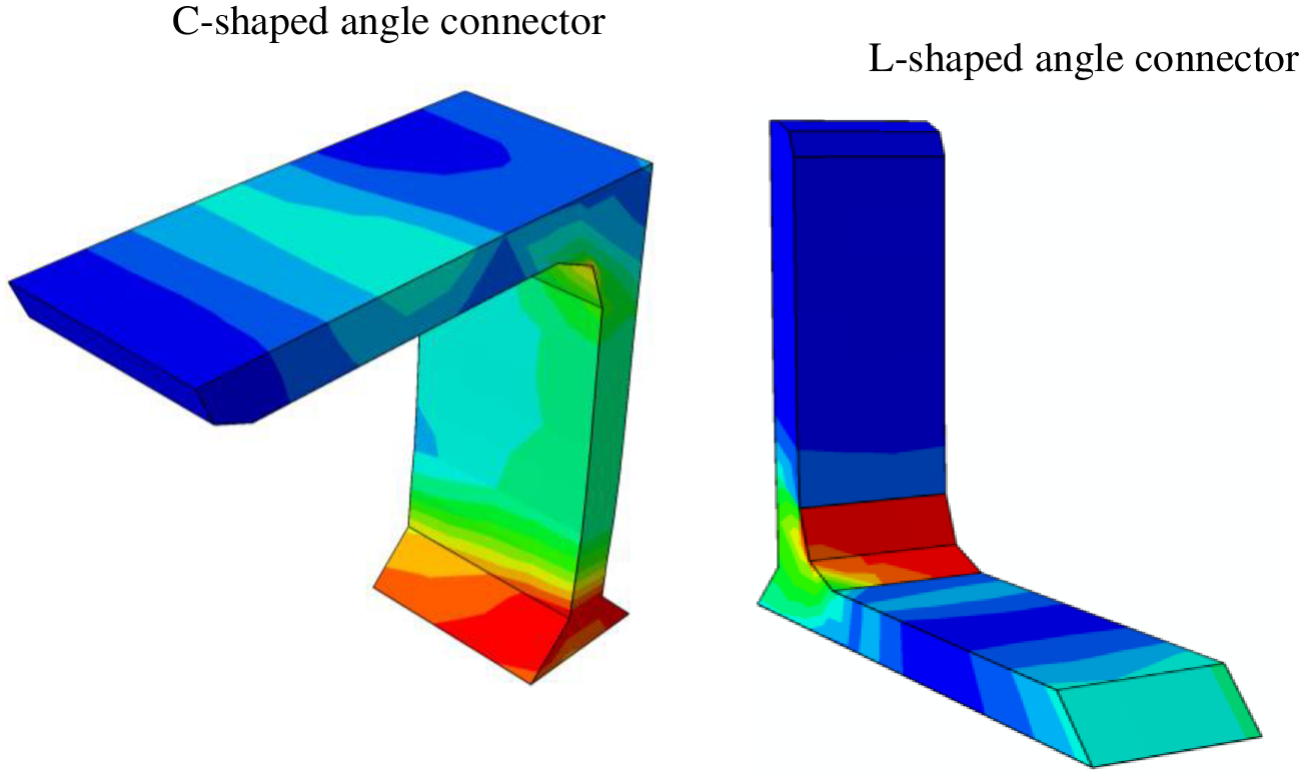
Stress contour of C-shaped and L-shaped angle shear connectors.

**Fig 20:**
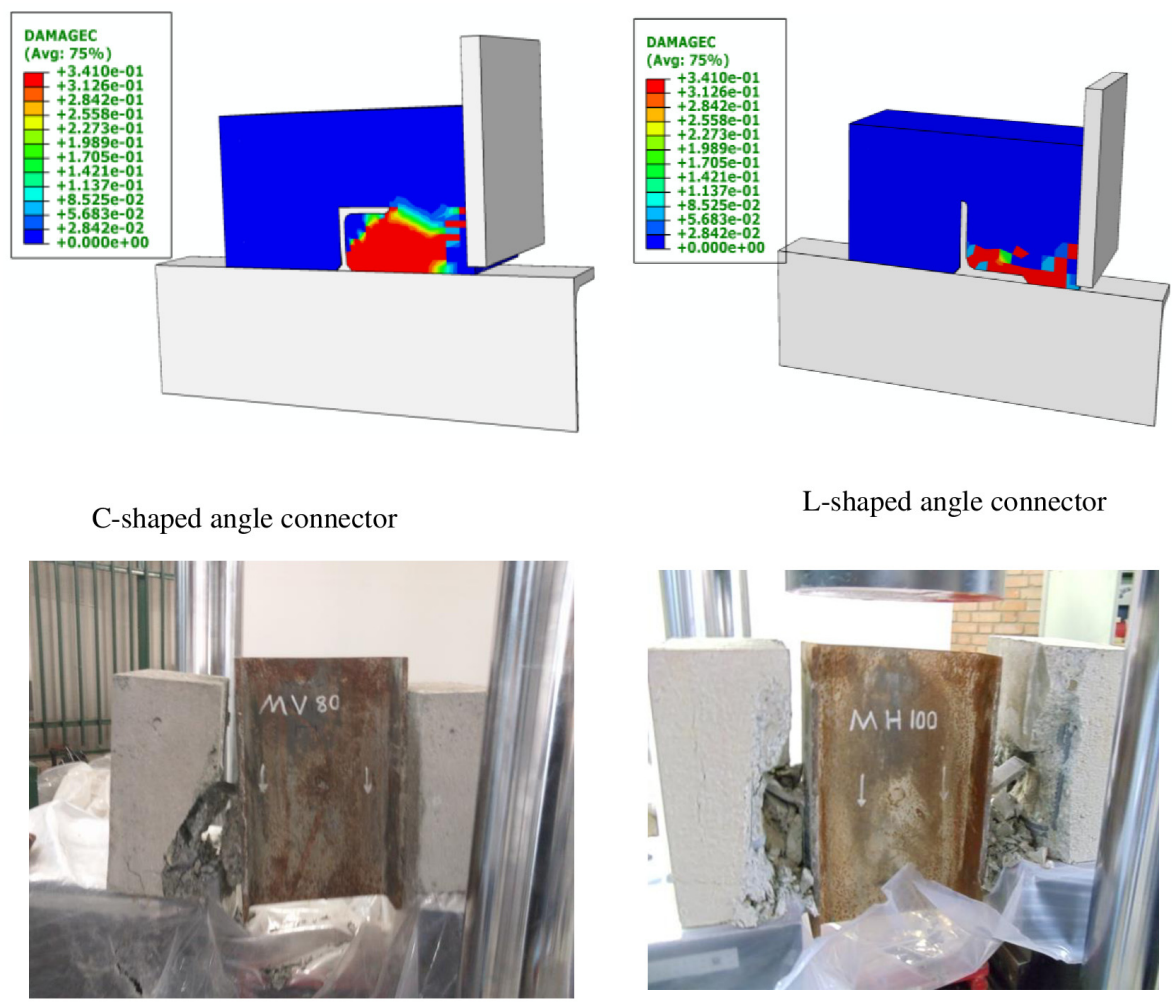
Comparison of concrete damage in FE analyses and the push-out tests.

The von Mises stress distribution, as obtained from the FE analysis for a C-shaped and L-shaped angle connectors are presented in Fig 18. It could be observed from this figure that the maximum stress in the C-shaped angle connector is taken place in the weld attachment to the beam and in the L-shaped connector is in the toe of the leg fillet. This is shown clearer in Fig 19 by extracting the angle part from the model. Fig 20 compares the concrete damage that takes place in the FE model against the actual test results for both types of the connectors. In all cases reasonable prediction is obtained from FE analyses.

### 5.2 Derivation of shear strength equation for C-shaped angle connectors

The finite element simulations are employed in order to investigate the effects of critical parameters such as compressive strength of concrete, thickness of angle shear connectors and the length of shear connectors on the shear strength of C-shaped connectors. The predicted equation is in the following form with an unknown coefficient C,

$$\text{Q}=\text{C}.{\text{L}}_{\text{c}}.\surd \text{t}\surd \text{f}{’}_{\text{c}}$$ (10)

The results of FE analyses of different connectors with varying parameters are presented in Table 3. The last column indicates the unknown coefficient needed for the above equation to be exact. Since the coefficient of variation for the data in the last column is only 0.08, the mean value of 0.213 is suggested for use in Eq 10.

**Table 3:** Result of finite element analyses for C-shaped angle connectors.

Specimen	h(mm)	f’_c_	t(mm)	L_c_(mm)	Q(kN)	$Lc\sqrt{t}\sqrt{f\prime_{c}}$	Coefficent
60×6	60	20	6	50	120.27	547.7226	0.2196
60×6	60	25	6	50	138.21	612.3724	0.2257
60×6	60	30	6	50	154.05	670.8204	0.2296
60×6	60	35	6	50	167.70	724.5688	0.2314
60×6	60	40	6	50	180.47	774.5967	0.2330
60×5	60	30	5	50	140.03	612.3724	0.2287
60×8	60	30	8	50	162.20	774.5967	0.2094
60×10	60	30	10	50	196.54	866.0254	0.2269
80×8	80	20	8	50	128.08	632.4555	0.2025
80×8	80	25	8	50	148.49	707.1068	0.2100
80×8	80	30	8	50	161.26	774.5967	0.2082
80×8	80	35	8	50	183.80	836.6600	0.2197
80×8	80	40	8	50	199.52	894.4272	0.2231
80×6	80	30	6	50	151.62	670.8204	0.2260
80×10	80	30	10	50	165.64	866.0254	0.1913
100×10	100	20	10	50	130.38	707.1068	0.1844
100×10	100	25	10	50	150.33	790.5694	0.1901
100×0	100	30	10	50	166.46	866.0254	0.1922
100×10	100	35	10	50	181.36	935.4143	0.1939
100×10	100	40	10	50	199.30	1000.0000	0.1993
100×6	100	30	6	50	165.98	670.8204	0.2474
100×8	100	30	8	50	176.70	774.5967	0.2281
60×6	60	30	6	30	90.13	402.4922	0.2239
60×6	60	30	6	80	215.10	1073.3126	0.2004
60×6	60	30	6	100	241.33	1341.6408	0.1799
						Average =	0.213

## 6. Conclusions

A finite element model was developed to simulate the load–displacement behaviour of the C-shaped and L-shaped angle shear connectors that were tested experimentally. The FE model takes into account the linear and nonlinear material properties of concrete and steel angle connector as well as the nonlinearity due to the contact conditions. Concrete damage plasticity was also included to better estimate the concrete’s behaviour after cracking. The model compared well with the test results and confirmed the conclusions that were derived from experimental test results. The FE analyses show that the peak stress in the connector happens in the attachment weld for the C-shaped connectors whereas for the L-shaped connectors, the critical stress is at the bottom of the connected leg. The concrete shows more damage in compression on the interior side of the angles in all cases.

Parametric studies using this model were carried out to investigate the effects of compressive strength of concrete, flange thickness and length of angle shear connectors on the ultimate shear strength of C-shaped connectors. Finally, an equation was suggested for predicting the shear strength of C-shaped angle shear connectors.

## Supporting Information

S1 File Comparison of the shear strength capacities of C-shaped angle connectors obtained from FE analyses and experiments. (XLSX)

S2 File Comparison of the shear strength capacities of L-shaped angle connectors obtained from FE analysis and experimental. (XLSX)
